# Externally validated models for first diagnosis and risk of progression of knee osteoarthritis

**DOI:** 10.1371/journal.pone.0270652

**Published:** 2022-07-01

**Authors:** Philippa Grace McCabe, Paulo Lisboa, Bill Baltzopoulos, Ivan Olier

**Affiliations:** 1 School of Computer Science and Mathematics, Liverpool John Moores University, Liverpool, England, United Kingdom; 2 Research Institute for Sport and Exercise Sciences, Liverpool John Moores University, Liverpool, England, United Kingdom; Public Library of Science, UNITED KINGDOM

## Abstract

**Objective:**

We develop and externally validate two models for use with radiological knee osteoarthritis. They consist of a diagnostic model for KOA and a prognostic model of time to onset of KOA. Model development and optimisation used data from the Osteoarthritis initiative (OAI) and external validation for both models was by application to data from the Multicenter Osteoarthritis Study (MOST).

**Materials and methods:**

The diagnostic model at first presentation comprises subjects in the OAI with and without KOA (n = 2006), modelling with multivariate logistic regression. The prognostic sample involves 5-year follow-up of subjects presenting without clinical KOA (n = 1155), with modelling with Cox regression. In both instances the models used training data sets of n = 1353 and 1002 subjects and optimisation used test data sets of n = 1354 and 1003. The external validation data sets for the diagnostic and prognostic models comprised n = 2006 and n = 1155 subjects respectively.

**Results:**

The classification performance of the diagnostic model on the test data has an AUC of 0.748 (0.721–0.774) and 0.670 (0.631–0.708) in external validation. The survival model has concordance scores for the OAI test set of 0.74 (0.7325–0.7439) and in external validation 0.72 (0.7190–0.7373). The survival approach stratified the population into two risk cohorts. The separation between the cohorts remains when the model is applied to the validation data.

**Discussion:**

The models produced are interpretable with app interfaces that implement nomograms. The apps may be used for stratification and for patient education over the impact of modifiable risk factors. The externally validated results, by application to data from a substantial prospective observational study, show the robustness of models for likelihood of presenting with KOA at an initial assessment based on risk factors identified by the OAI protocol and stratification of risk for developing KOA in the next five years.

**Conclusion:**

Modelling clinical KOA from OAI data validates well for the MOST data set. Both risk models identified key factors for differentiation of the target population from commonly available variables. With this analysis there is potential to improve clinical management of patients.

## Introduction

### Background and significance

Osteoarthritis (OA) is a degenerative bone disease that affects joints as a whole and is one of the most common diseases affecting people in old age. The prevalence in people 65 years and older ranges from 12% to 30% [1]. The condition is also the most common form of arthritis to cause pain and mobility limitations. OA most commonly affects the knee, and around 10% of people over 55 years old have knee OA (KOA). This value is not surprising as weight-bearing joints, such as the knee or hip, are where disease occurs most [2]. The focus of this paper is specifically KOA.

According to a review conducted in 2020 the third largest area of UK National Health Service (NHS) spending in 2013–2014 was musculoskeletal conditions, including OA costing £4.7 billion [3–5] with the cost only expected to rise as the population ages. By 2017 the total cost of OA and rheumatoid arthritis in any joint on the NHS and the wider healthcare system was £10.2 billion [6]. As more people develop the disease, the costs attributed are going to increase and put further strain on the NHS. In the UK in 2017 there were 120,581 knee replacements and OA was the primary cause for 98% of these [6]. Therefore, there is a clear and definite need for diagnostic aids and models to indicate risk of disease onset and progression as currently there are no modelling tools in use.

There is more than one way to define KOA. One commonly used approach is the Kellgren and Lawrence (KL) grade, which was accepted by the WHO in 1961 [7]. There are five stages of KOA according to the KL scale [8]. These are differentiated between with the use of x-rays to determine the severity of the OA. Stage 0 is classed as no OA and Stage 4 is severe OA present in the joint. For the purpose of the study described in this paper a score of KL 0 or KL1 means no KOA, and a score of KL 2 or higher means KOA is present.

The work in this paper would provide the base for a model that could be utilised as a screening tool. This would be useful as having a filter to help determine what candidates require further investigations, such as x-rays, would help to reduce the cost of diagnosis, and potentially help to speed up diagnosis, delay disease progression, improving the process from a patient perspective. The main clinical problem is two-fold. Firstly, there is a need to determine who has KOA at their first presentation to a clinician. Then, of those without the disease, establish who is likely to progress to KOA after a period. By identifying those with the disease, it becomes possible to indicate which subjects require interventions, such as more frequent follow-ups, to assess how the disease is affecting them, improving the patient experience. Similarly, by highlighting individuals at risk of developing the disease it would be possible to offer actions that may allow for a reduction in risk of early onset. This may be help to lose weight, reducing the BMI of an individual, taking them from a high risk to a low-risk group for developing KOA in a five-year timeframe.

Machine learning (ML) models are used in a wide variety of application domains, including healthcare. By leveraging ML within tools for clinical application it becomes possible to use a wider variety of input information to optimise the decisions about patient care in a way that would be very time intensive for a clinician to do themselves. Furthermore, the models could help to streamline the way clinical decisions are made, such as in OA, by providing a suggested course of action and helping to eliminate clinician bias.

Methods are typically put into place for experts in the application area to interpret and understand the results in a way that is intuitive. In the real world, for areas that ML methods are being used it is critical for the successful and appropriate implementation and safe crossover that mathematical algorithms that are used in decision-making processes are capable of being integrated into human reasoning models. Crucially, the provision of interpretation for AI is now arguably a central pillar for the “right to explanation” written into the General Data Protection Regulations (GDPR) which came into force on 25^th^ May 2018 [9].

In recent years there have been several risk prediction models developed that aim to identify the risk factors that can indicate the presence of KOA. Models, such as those produced by Zhang et al attempt to identify the presence of radiographic or symptomatic KOA [10], and these models focus on the use of logistic regression with varying pools of variables. It is worth noting that many modelling techniques have been used when attempting to develop prediction tools for KOA including support vector machines, tree based methods, mixed-effect mixture models and modelling using MRI data [11]. As models for KOA have great potential for use in clinical settings more complex methods are also commonly used [12–14]. As with any approach to ML modelling, there are advantages and limitations. In the work by Sheng et al the modelling was carried out with Bayesian networks (BN) which are useful as they have the advantage of being able to learn any pattern present in the data. The leading drawback of the BN approach is that there is no universally accepted method to model the network, which results in a time intensive process. The model also requires the user to influence the features that will be used in the network but the advantage to this is that unlike neural networks, it is possible to ensure domain specific information is included. Another method that was used is the artificial neural network (ANN) in the paper by Yoo et al [12]. ANNs are growing in popularity and are used in a variety of application domains, including healthcare. ANNs have the advantage of being able to handle a large amount of input features and non-linear relationships in the data. The drawback to ANNs comes from the computation expense to run the model and there black box nature, which makes the results hard to interpret to something meaningful. Another popular method in prediction modelling, and is used in the paper by Widera et al is random forests (RF). In a similar way to ANNs the main disadvantages to RF are the lack of interpretability and that the models are computationally expensive to run. The RF models have the advantage of being able to handle both linear and non-linear data well with minimal pre-processing. However, there is a pattern that logistic regression is the preferred modelling method for risk prediction analysis due to its ease of interpretation and long standing use within the medical domain.

Other models that have been developed using the OAI and MOST datasets typically either use a similar variable set and different approaches, such as the model described by Losina et al [15], or make use of the extensive MRI and x-ray data available from both studies, such as the work by Tiulpin et al [16]. Although the model by Losina et al is similar to the one described in this paper it is fundamentally different. The paper based on the OAPol model, which considers comorbidities such as cardiovascular disease and cancer is a Monte Carlo model running simulations, based on transition probabilities from literature produces risk percentages for the likelihood of an individual to develop KOA or require total knee replacement in windows of time ranging from 5 years to lifetime. The model in this paper uses variables known to be related to KOA onset, and use standard approaches, such as logistic and Cox regression to produce diagnostic and predictive model. The paper from Tiulpin et al offers additional information from leveraging x-ray images to assign a KL grade to determine the presence of KOA in an individual. However, our model uses only clinical features to ascertain if a person has the disease, which is of benefit when the subject does not yet have a knee x-ray, which is often the case in primary care settings.

When considering models for use with diseases such as KOA there are often many issues, spanning from data availability to problems with reported values due to differing interpretation of questions. Often, with disorders such as KOA, when recruiting participants the pool of subjects is limited, resulting in small sample sizes for modelling [13, 17]. Another issue is that subjects often do not have a formal diagnosis and therefore the modelling outcome can be self-reported [13]. A 2017 study developed a risk prediction model to predict knee pain, but stated this could not be extended to the use of KOA [18]. The study by Fernandes et al provides an example of a tool that may be used in a primary care setting as a way to anticipate future problems. Although this study cannot be extended to KOA due to a lack of available information, it does help to cement the potential usefulness of the models within primary care [18].

Survival analysis models, if used in clinical settings, could be useful for patient education by quantifying the risk of progression over time, particularly in response to modifiable risk factors. One model used the Swedish conscription registry to model the 40-year risk of developing KOA in males, which showed promising results, with reported AUC values between 0.6 and 0.7 in the study population, for the application of this type of model to be used in the wider population [19]. However, this model only focuses on males, which may influence the model discrimination when applied to female subjects, and uses logistic regression, instead of survival specific approaches, such as Cox regression. Similarly, a 4 year risk model was produced that used logistic regression, but lacked external validation [20].

There is also a scarcity of longitudinal models of KOA so the objective in our study was to develop and validate a diagnostic and prognostic model to determine the presence of KOA at baseline or to calculate if a subject is at high or low risk of developing KOA in the next five years. The models can then be converted into web apps that have the potential to be used in clinical settings, such as GP surgeries to help streamline both diagnosis and patient education, leading to better clinical management and self-perceived quality of life for those with KOA. By having a tool available that a GP could use in a standard clinical visit, a few additional questions could provide further insight to the patient relating to their risk profile of having KOA, or their risk of developing the disease. For example, visits to a GP for unrelated health issues in a person above a certain age already triggers a flag to be asked screening questions for other diseases, so this one could be used in the same way, educating the patient about their risk for developing KOA. The diagnostic model could be used for those reporting knee problems as a way to help signpost to further interventions.

Although Joseph et al. [21] produced a model that is an app, the inputs require information from MRI images and focused on those with none or mild KOA determined from an x-ray. A model that relies on MRI or x-ray information can provide further insight into diagnostic and prognostic decisions; however, for use at a primary care setting where this information is not available a model that can provide decisions without is imperative. Our study used features that are gathered solely from the subject in any person aged 45 or over to determine their individual risk of having KOA. This could then be used to determine if the person required further interventions, such as x-rays or MRI scans to definitely confirm this determination.

The survival model could emphasise the need for someone to change their weight in order to reduce their risk of developing KOA in the next five years. This would require a modification into the life of the subject. In order for an intervention to work, there needs to be some level of subject acceptability, referring to the suitability of the intervention to both those delivering and receiving the care [22]. When trying to implement change into the way a subject behaves based on a potential outcome then a nearer end-point can be seen as more advantageous. A similar approach is used when trying to encourage people to quit smoking, displaying relatively short time steps into the future, given with the associated benefit [23]. One such example is that 48 hours after quitting smoking a person’s taste and smell receptors begin to heal [24].

We apply Cox regression to take into account recorded time of onset, which is one of the novel aspects of this paper. We also use less conventional risk factors in our model for diagnosis, such as difficulty getting upstairs. Variables such as this are useful as they consider the holistic impact as a result of potential KOA, with other models instead using measures relating to imaging. This is self-reported, which makes it easy to acquire, but it turned out to attain statistical significance in our models. Both the diagnostic and prognostic models are also externally validated.

We have identified a gap in the literature relating to KOA diagnosis and prediction. In the first instance, for knee OA there is not a specific unified framework for identifying KOA, and the diagnosis model in this paper builds on the existing knowledge that is used in clinical practice. Currently, the only prognostic models available for KOA are for determining time from diagnosis to intervention, such as a knee replacement. The model described in this paper looks at an at-risk individual and calculates the risk of disease in the next 5 years given that at present they do not have the disease.

## Methods

### Data sources

For the modelling and validation, two datasets were required. We have used the Osteoarthritis Initiative (OAI) where the data is available via the NIMH data archive at https://nda.nih.gov/oai/ and the Multicenter Osteoarthritis Study (MOST), where data is available from the University of California San Francisco (https://most.ucsf.edu/).

#### Osteoarthritis Initiative (OAI)

The Osteoarthritis Initiative was a multi-centre study, conducted over a 10-year period in America starting in 2004, is initially made up of 4796 subjects, aged 45–79 who were recruited based on their likelihood to develop knee OA [25]. In the OAI study, clinical examinations, questionnaires and telephone interviews were conducted at varying intervals and the results recorded. In the analysis described in this paper, the data that is used is collected from questionnaires that the subject completed and the KL score, used as the indicator for presence of KOA, collected from the clinician analysing the x-rays. For the features used in the models in this analysis only data recorded at the initial visit was required as inputs into the model and the diagnostic KL grade, with the KL grade from follow-up visits being used for the time-to-event analysis. The data tables used in these analyses are AllClinical00, which combines data about subject characteristics, risk factors and medical history, and kxr_sq_bu00, kxr_sq_bu01, kxr_sq_bu03 and kxr_sq_bu05, which contain information relating to the clinically assessed x-rays at baseline, first, second and third follow up respectively. The main variables are taken and adapted from AllClinical00, whilst the outcome for the diagnostic model are from kxr_sq_bu00 and outcomes and times for the survival modelling are from the remaining tables.

For the diagnostic modelling, the original cohort had a sample size of 4796. After reducing the sample by removing those who have no KL grade a sample of 4507 subjects remained. Finally, removing those subjects who have missing values in any portion of the variable sets leaves a usable cohort of 2707 subjects in the complete case analysis.

For the prognostic modelling only subjects with no baseline KOA and at least one follow up measurement could be included, as this approach considers the time to change state from no disease to active KOA. Removing any subjects that had KOA at the baseline assessment and did not meet the follow-up filter leaves a sample of 2314 subjects. These subjects had no OA, in other words, a KL score of 0 or 1 at baseline. Considering basic demographic features for subjects where there are no missing values, the usable subject cohort is comprised of 2136 subjects. Filtering out any subjects with the event of interest outside of the 5 year cut off results in a sample size of 2005 subjects.

#### Multicenter Osteoarthritis Study (MOST)

The Multicenter Osteoarthritis Study (MOST) is a longitudinal, prospective, observational study of knee OA in older Americans with either pre-existing OA or at increased risk of developing it, with the data coming from two separate clinical centres [26]. The MOST dataset enrolled 3,026 study participants aged between 50 and 79 drawn from the general population and conducted five follow-ups. At each follow-up X-rays were collected, except for month 72. The data tables used from the MOST study are V0ENROLL and V01235XRAY.

The data from MOST for the diagnostic model validation was prepared in the same way as for the OAI data. All subjects were required to have no missing values for the variables present and an initial KL grade from the baseline assessment. The only difference for the MOST data is that one variable, knee_swell, was not present in the dataset. Therefore, to establish predictions from this data including this covariate, we marginalised over the other variable combinations and produced predictions.

Although logistic regression is linear in the parameters, we chose to discretise variables such as age and BMI in order to maximise the performance of the model since discretisation introduces new parameters (betas) which make the model piecewise linear. For instance in the case of Age, since this variable is skewed, the non-linearity is likely to be important.

### Data pre-processing

The type of data used in this analysis combines clinical factors, demographic features, self-reported symptoms and self-reported physical activity data. The clinical and demographic variables include the age, gender and BMI of the individual, along with information of family history and previous injuries to the knee. The self-reported data set comprises subject’s answers to questionnaires relating to their symptoms and how they are impacted, recorded at the first presentation meeting. In a similar approach to the self-reported features, the self-reported physical activity data set consists of answers on questions about how much exercise they take and how this impacts them.

For several features in the original data, more than one column is relevant. To streamline the analysis, and future usability in a clinical setting, we have taken the approach of defining new variables that incorporate the existing ones in a single feature. One such example is for the created variable knee_stiff_day_limit. This looks at how many days in the past 30 a subject has experienced knee stiffness severe enough to limit activity. Several original variables looked at various activities individually, so this approach removes repetition by taking the most severe measure for a subject across all variants of activity. In this situation, if a single variant contains a missing value, the present values are the only ones considered. If all are missing, the consolidated variable is also recorded as missing.

The cohort considered in this analysis was only subjects without any missing values for the selected variable set. This is a complete case analysis [27]. In the preliminary steps of the analysis, not detailed in this report, a complete case and imputed analysis were used and compared.

#### Variable selection

The diagnostic modelling uses variables considered to be relevant following literature reviews of similar analysis [10, 17, 21]. We know from the literature that features such as gender, genetic disposition, BMI and history of injury are all factors that contribute to the onset of KOA [28]. The decision was made to only include variables where data could be gathered from the subject alone without the need for additional testing. This involves excluding image data, such as x-ray or MRI data or performing additional testing like movement measurements. The variables used to consider the future development of the disease differ slightly from those to detect the disease at the point of medical intervention. Seventeen variables fitting clinical and demographic features were identified using the extracted OAI data. The variables include the age, gender and BMI of the individual, along with information of family history, previous injuries and diagnoses of osteoarthritis in other joints and general arthritis in the body. Several variables in the OAI data are self-reported. The self-reported data is made up from subject’s answers to questionnaires relating to their symptoms and how they are impacted, recorded at the first presentation meeting, along with data made up of answers on questions about how much they take exercise and how this impacts them. An initial analysis looking at only the clinical and demographic work was conducted, and the subsequently presented at IDEAL 2019, showing the idea for the diagnostic model [29].

The justification for the inclusion of features in both the diagnostic and prognostic model revolve around a known risk to KOA. The risk of KOA increases as age increases, similar to BMI, both of which are present in the diagnostic model, and only BMI used in the prognostic model. Gender was another feature with a clear link, such that females are more at risk of KOA than males of the same profile, resulting in this variable being used in both models. Stiffness and swelling are known symptoms that can indicate the presence of KOA, resulting in these features being used in the diagnostic model. Mobility was considered in the diagnostic model in the form of difficulty getting upstairs and knee pain that limited activity in the prior 30 days. A reduction in mobility is an indication of increased risk of KOA. Family history of OA was included in the prognostic model as the potential to indicate a genetic link for future development of KOA. It was important to consider injury when considering future risk of KOA resulting in the variables for ‘ever injured knee’ and ‘history of falling’ as the former indicates a known risk whilst the latter may suggest a higher likelihood for injury, increasing the risk for developing KOA in the future. Finally, WOMAC was used in the prognostic model as it provides the self-perceived view of the condition from the subject’s perspective, proving an indicator into how they feel at that time which may influence how that individual behaves in the future. For example, a high WOMAC score indicates a poor self-perceived view of the condition, possibly providing insight into how the person feels in areas of their life not covered by the other features.

#### Class definition

Clinical KOA in this analysis is a binary outcome defined by the KL score. These KL grades have been determined by a clinician from analysing the X-rays taken as part of the study. Scores zero and one are classified as no clinical KOA, and therefore zero. A KL score of two or above determines the positive class, clinical KOA, therefore classified as one as the binary indicator. For the survival modelling, presence of disease is noted in instances where a subjects disease state changes from that of no KOA to KOA, using the same KL criteria as described.

### Implementation

#### Experimental set-up

Logistic regression and Cox regression were optimised with AIC calculated from the test data. All data pre-processing, analysis and subsequent app construction were implemented in R. The logistic regression model uses the built in functions for the analysis in base R. For the prognostic modelling the packages used are survival [30], survAUC [31] and survminer [32]. The example web-based application was implemented with the shiny [33] package, a Web application framework for R.

#### Measure of performance

The receiver operating characteristic curve (ROC curve) is a plot that graphically indicates the ability of a model to correctly classify binary outcomes as a threshold is altered. The area under the curve (AUC) is equal to the probability that a classifier will rank a random positive instance higher than a randomly chosen negative one [34]. In the AUC a value of 0.5 indicates a guess, with greater than this being deemed better than a guess, and lower than 0.5 being worse than a guess. The AUC and confidence intervals are calculated using the package pROC [35]. The AUC is calculated using the trapezoidal rule and the 95% confidence interval using 2000 stratified bootstrap replicates.

Sensitivity, specificity and positive predictive value (PPV) are all statistical measures of the performance of binary classification tests. The sensitivity measures the proportion of actual positives that are correctly identified. The specificity measures the proportion of actual negatives correctly identified. The PPV measure looks at the amount of correctly classified subjects out of the whole group of disease class predictions. In this analysis, these measures are calculated with the caret package [36]. Sensitivity is the true positive rate; it is an indicator of how likely a model is to correctly identify a patient with a disease. If a model has high sensitivity it can help to rule out a disease where a person is not indicated to have the disease. Specificity is another measure assessing the way a model performs, this time indicating the true negative rate. This is a way determine how effectively a model can correctly identify people without a disease. Models with high specificity can be used to rule in disease in a person who is indicated to have said disease, potentially prompting further investigation. Positive predictive value, (PPV), is the odds of having the disease if you have a positive result. This measure is useful to both the patient and clinician as it can be used in conjunction with sensitivity to indicate how likely a positive result is actually true.

The methods used for assessing the model performance have been chosen as they are already widely used and understood in clinical applications. The sensitivity, specificity and PPV are all useful when considering models for clinical use as they have real meaning to both clinician and patient. The AUROC curve is a good model for assessing two class problems, like the one for the diagnostic model. The larger a value of AUC the better the model performs overall, leading to an easy interpretation of how suitable a model will be for implementation as a ‘rule-out’ test in a clinical setting [37, 38].

#### Approaches to modelling techniques

Given a longitudinal dataset, it is possible to conduct both diagnostic and prognostic modelling. A diagnostic model using logistic regression is the initial model for determining, at baseline presentation, if a subject has KOA. In the population without KOA at baseline, the focus shifts from identifying the disease to predicting the time to onset. Survival analysis for time to event models is a standard approach when modelling cases in those initially without disease. By using a baseline set of predictor values it is possible to determine an individual’s risk for developing KOA in a five year follow-up. Cox regression is the standard approach for modelling when using current information to make predictions about the future.

#### Diagnostic model

The model used in the diagnostic analysis was logistic regression. The logistic regression approach was chosen after considering alternative analysis methods [29]. This method is preferred by clinicians as it mimics their own decision making process. The goal for the logistic model is to determine, based on eight features relating to a subject, whether they are likely to have KOA and therefore require further investigations into their symptoms.

The presence of clinical KOA, KL grade 2 or above is the outcome. The model was trained and tested using the OAI data with 1353 and 1354 subjects respectively.

Each of the variables can be represented as switches that are either on or off, or contribute on a sliding scale to the overall outcome. This is useful for when trying to predict a disease outcome as these decisions are never black and white, but more often than not when based solely on symptoms come with a scale of how much the covariates contribute to the overall outcome.

#### Prognostic model

The prognostic analysis uses Cox regression to model how the covariates jointly influences the probability of the subject developing KOA. After modelling with Cox, we created cohorts by risk stratification, to highlight the criteria for being at low and high risk for developing KOA in 5 years from the baseline assessment.

The groups used to model this analysis are taken from the original OAI data but removing subjects with KOA at their initial assessment. The model was then trained and tested on 1002 and 1003 subjects from the OAI dataset respectively.

To stratify the group into cohorts the first step is to establish a cut-point that provides the largest separation between subjects. This is done with a model containing five variables from the subjects’ initial assessment. The Cox model produces predictions of the risk score for the training dataset. These are plotted into a histogram, displaying the distribution of risk scores, showing a Chi^2^ distribution. The predictions are shown in bins of 0.25 from 0 to the maximum value calculated wising the original Cox model.

At each bin interval for the risk score the values above the cut point are assigned to cohort 1 and those below are assigned to cohort 2. Using the cohorts, a Cox model is fitted to the data and two baseline hazards are fit to the strata. The next step is to test the strata to see if there is a difference between the curves with a log rank test. This test produces a Chi^2^ statistic, which is used to determine the p-value relating to the cohort stratification. This is repeated for each bin interval of the risk score, storing the p-values at each iteration. To identify the risk score that relates to the optimal cut point for the cohorts the minimum p-value is determined and the corresponding risk score is selected. This is then the score used to split the population into two cohorts.

#### External validation

To validate and ensure that these models were not overfitting to the OAI data used to train them, we used the MOST data to validate the results. The MOST data was collected from different centres than those used in the OAI study so this helps to determine if the model is able to avoid institutional bias. This helps to assess if the model can be used outside of the bounds from which the data was collected, and also contributes to showing that a prediction model is more suitable for use in clinical practice [39].

The validation set for the diagnostic model is 2006 subjects, whilst the validation set is 1155 subjects.

**Fig 1:**
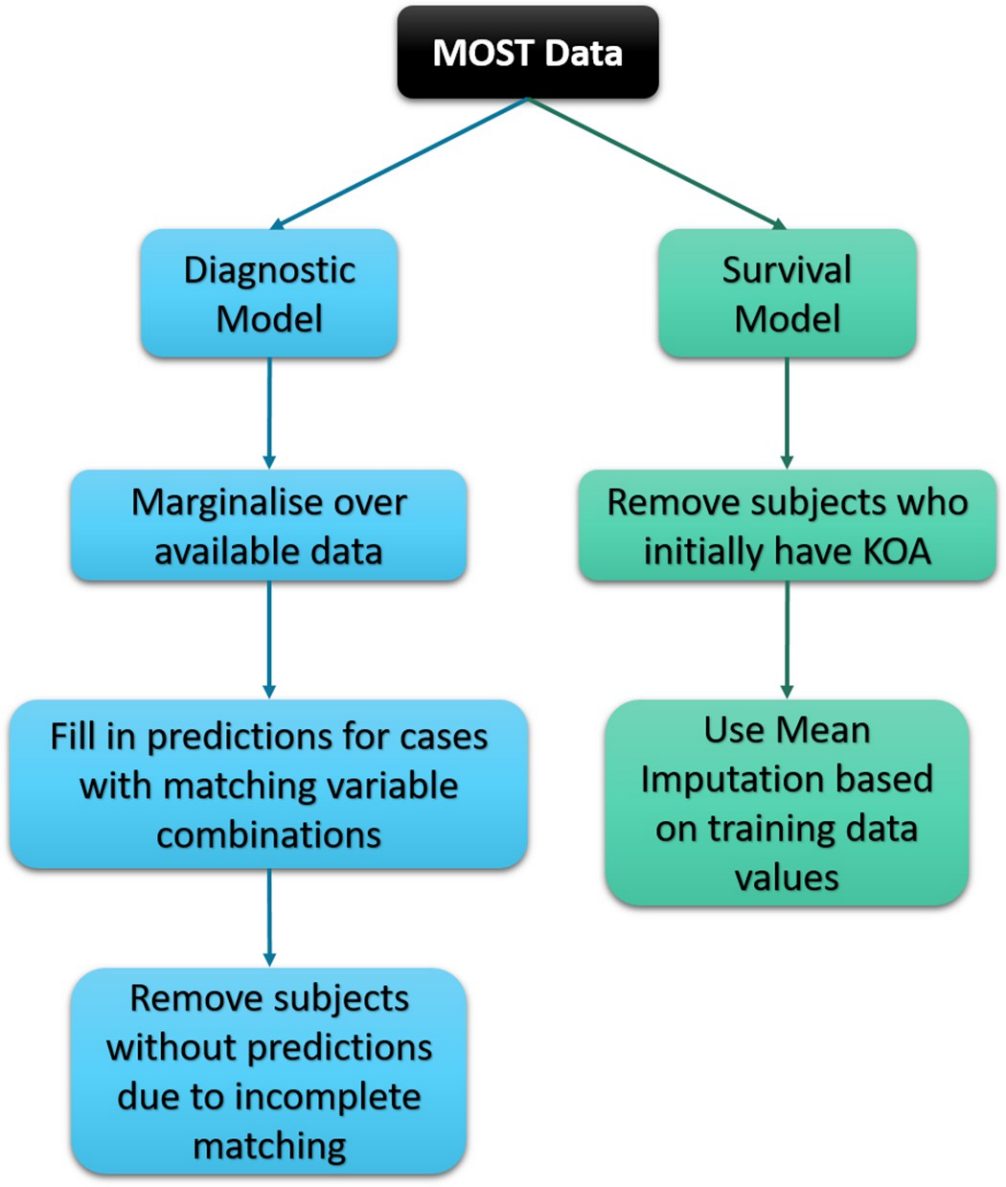
Missing data flowchart. A flowchart for how the missing values are dealt with in the MOST validation data.

The data used to validate the models contained missing values. The approaches to dealing with differed depending on the model. A visual representation of how the missing values were accounted for are shown in Fig 1.

For the diagnostic model the variable knee_swell was missing from the MOST dataset. To combat this we marginalised over the existing variable combinations and produced predictions based on those from the OAI training data. After doing this, some samples were lost due to incomplete matching where the variable combinations in the MOST data did not have a corresponding combination in the OAI data that was used in the marginalisation. As a result, the sample size is reduced from 2006 to 831 subjects.

For the prognostic model validation, again those with KOA at baseline were removed from the analysis. Date filtering also removed subjects whose event fell outside of the 5-year cut-off. To fill in the missing values to ensure a sufficiently sized dataset mean imputation based on the training data was used. For the imputation in the MOST data, the family history imputation is ‘No’, along with the history of falling. The imputation for the WOMAC score given as a value of 8. This approach resulted in a sample size of 1155 subjects. The WOMAC score is the Western Ontario and McMaster Universities Arthritis Index that is used to assess hip and KOA, measuring items related to pain, stiffness and physical function. The questionnaire has a total score range of 0–96, with higher scores indicating a more severe outcome.

## Results

The OAI training data was used to develop the models and the MOST data was used as external validation. The OAI data training and test sets have a prevalence of KOA at 40% and 39% respectively. The MOST validation set prevalence is at 60%. The probabilistic cut-off for binary classification was taken to be 0.5. The variables in the diagnostic model are Age, BMI, Baseline Symptoms, Gender, Knee pain affecting activity in the past 30 days, Difficulty getting upstairs and Knee stiffness, with summary statistics shown in Table 1. The variables for the prognostic model consist of BMI, family history, ever injured knee, history of falling, gender and WOMAC score, with the summary statistics detailed in Table 2.

**Table 1:** Diagnostic model summary information of the Osteoarthritis Initiative (OAI) and Multicentre Osteoarthritis Study (MOST) datasets.

Diagnostic Model Data					
Variable		OAI			MOST
		Total *N = 2707*	Training Set *n = 1353*	Test Set *n = 1354*	*N = 2006 After Marginalisation n = 831*
**BMI**	Less than or equal to 25	754	383	371	792 (68)
	More than 25	1953	970	983	1214 (763)
**Baseline Symptoms**	No	2042	1028	1014	439 (139)
	Yes	665	325	340	1567 (692)
**Knee pain affecting activity in the past 30 days**	No	2068	1031	1037	167 (142)
	Yes	639	322	317	1839 (689)
**Knee swelling**	No	1970	993	977	
	Yes	737	360	377	
**Gender**	Male	1250	622	628	742 (298)
	Female	1457	731	726	1264 (533)
**Difficulty Upstairs**	No	1352	660	692	136 (30)
	Yes	1355	693	662	1870 (801)
**Age**	45–50 years	373	184	189	108 (45)
	50–55 years	572	281	291	416 (198)
	55–60 years	457	232	225	350 (151)
	60–65 years	402	195	207	390 (166)
	65 years or over	903	461	442	742 (271)
**Knee Stiffness in the past 30 days**	0 days	2069	1032	1037	1242 (144)
	1–7 days	289	152	137	266 (251)
	8–14 days	118	59	59	76 (55)
	15–21 days	114	58	56	104 (91)
	22 days or more	117	52	65	318 (290)
**KL status**	KL < 2	1627	806	821	792 (272)
	KL 2+	1080	547	533	1214 (559)

The variables are listed with the different options each can take. As the knee swelling data is missing in the MOST dataset, the predictions are marginalised over the OAI data to find outcomes that match the cases for the other variable combinations. The number in brackets represents the number per variable after marginalisation has taken place.

**Table 2:** Prognostic model summary information of the Osteoarthritis Initiative (OAI) and Multicentre Osteoarthritis Study (MOST) datasets.

Prognostic Model Data					
Variable		OAI			MOST *N = 1155*
		Total *N = 2005*	Training Set *n = 1002*	Test Set *n = 1003*	
**BMI**	Less than 25	644	329	315	234
	25–29.9	814	409	405	478
	30+	547	264	283	443
**Family History**	No	1601	800	801	328 [352]
	Yes	404	202	202	475
**Ever Injured Knee**	No	1239	621	618	671
	Yes	766	381	385	484
**History of Falling**	No	1341	624	717	130 [1003]
	Yes	664	378	286	22
**Gender**	Male	895	373	522	693
	Female	1110	629	481	462
**WOMAC**		0–82 (6.8)	0–71 (8)	0–82 (5)	0–82 (10) [4 *8*]
**KOA**	Censored	1839	913	926	1004
	Develop KOA	166	89	77	151

The OAI training data was used to develop the models and the MOST data was used as external validation. The variables are listed with the different options each can take. To have a meaningfully sized dataset NA values are present in the MOST cohort. They are only present in three variables, with the amounts shown in square brackets next to the value they are imputed to. The family history imputation is ‘No’, along with the history of falling. The imputation for the WOMAC score is shown in italics, with a value of 8.

The odds ratios, modelled on the OAI data, described in Table 3, are against the reference category for each variable. These are namely no knee pain exhibited on the day of the baseline assessment (B.Line_SYMP), participants aged 45–50 (AGE_bins), any subject whose BMI was 25 and over (BMI_bins), male participants (Gender), no difficulty in getting up stairs (Diff_Upstr), not modifying activity from knee pain in the past 30 days (KPACT30) and 0 days of stiffness in the past 30 (Knee_Stiff). Within Table 3 the odds ratios for knee_stiff are close to zero, meaning that the variable does not contribute significantly to the outcome. The large confidence intervals show that the variable is not a significant contributing factor to the likelihood of having KOA at the point of first presentation.

**Table 3:** Coefficients of logistic regression.

	Odds ratio	95% CI
		Lower—Upper bound
**Intercept**	0.26	-0.14–0.66
**Age 50–55**	1.11	0.67–1.55
**Age 55–60**	1.63	1.18–2.07
**Age 60–65**	2.01	1.55–2.47
**Age 65+**	2.21	1.81–2.61
**BMI less than 25**	0.51	0.22–0.79
**B.line_symp yes**	4.80	4.49–5.10
**Gender female**	1.32	1.07–1.57
**KPACT30 yes**	> 100	> 100–>100
**Diff_upstr yes**	1.10	0.84–1.36
**Knee_stiff 1–7 days of stiffness**	~0.00^a^	-636.50–636.50
**Knee_stiff 8–14 days of stiffness**	~0.00^a^	-636.50–636.50
**Knee_stiff 15–21 days of stiffness**	~0.00^a^	-636.50–636.50
**Knee_stiff 21+ days of stiffness**	~0.00^b^	-636.50–636.50

^a^The values for are 0.000001, therefore approximate to 0.00 to 2 decimal places

^b^ The values for are 0.000002, therefore approximate to 0.00 to 2 decimal places

**Fig 2:**
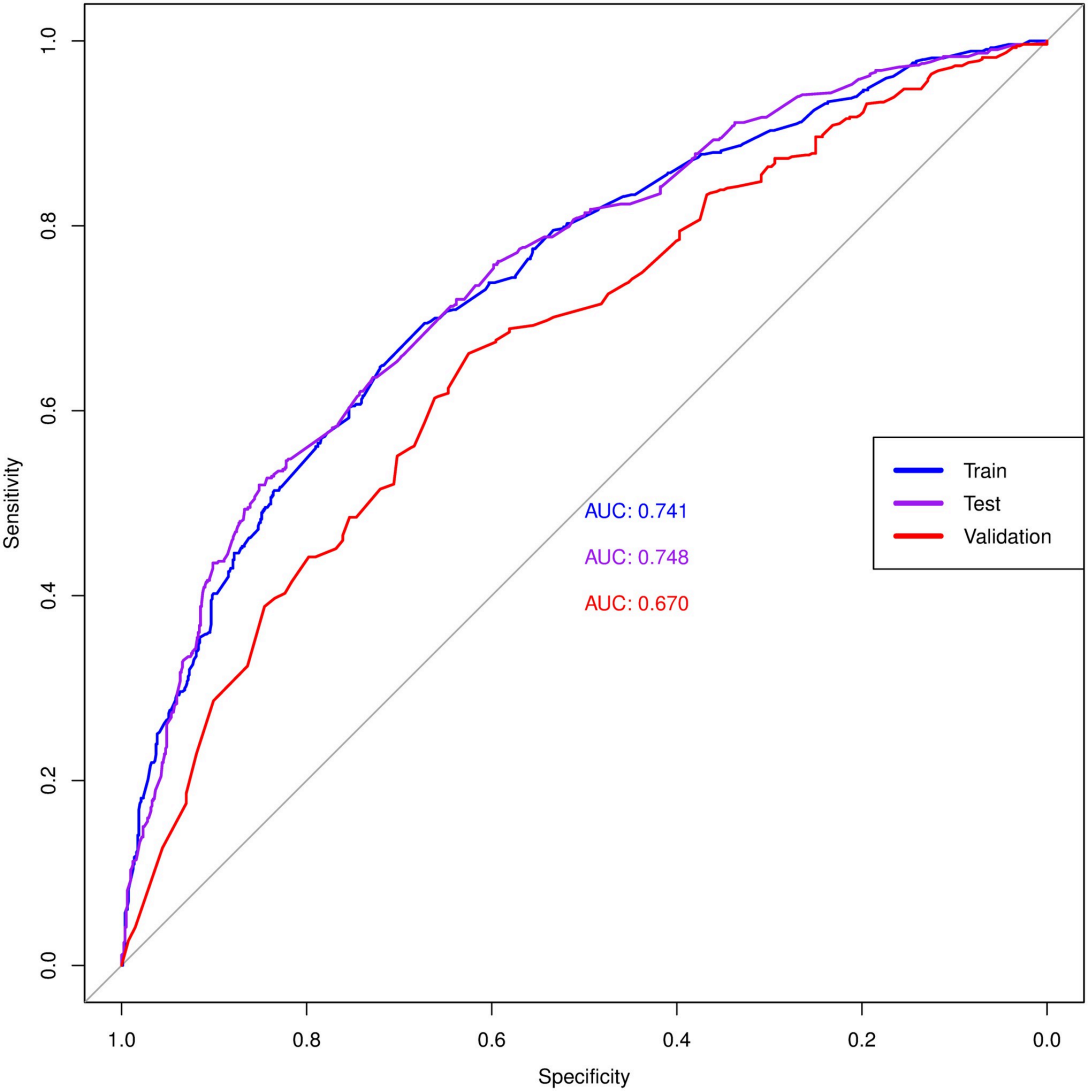
ROC curves. ROC curves for the OAI training and test models, and the MOST validation model. The AUC for each curve is listed on the curve.

Summary statistics for the diagnostic model, for test and validation are listed in Table 4 and the ROC curves are in Fig 2. The interesting results are that the MOST data performs quite well on a model developed using the OAI data. The MOST results have a high sensitivity, meaning that the model identifies about 90% of all KOA cases. To assess how the model performed using the MOST validation data when compared to the OAI data, the AUC and 95% CI were compared. Although the 95% CI does not overlap for the OAI and MOST data, the difference between is 0.64%, therefore with a slightly larger sample size, or all variables available in the MOST data there is the possibility that the CI values would overlap for the two datasets. Therefore, modelling clinical KOA from OAI data validates well for the MOST data set.

**Table 4:** Summary statistics for the diagnostic model.

Measure	OAI—Training	OAI—Test	MOST VALIDATION
Sensitivity	0.4790	0.5197	0.9052
Specificity	0.8511	0.8490	0.2353
PPV	0.7629	0.7748	0.5421
AUC	0.7415	0.7475	0.6697
(95% CI)	(0.7146–0.7683)	(0.7209–0.7742)	(0.6311–0.7082)

**Fig 3:**
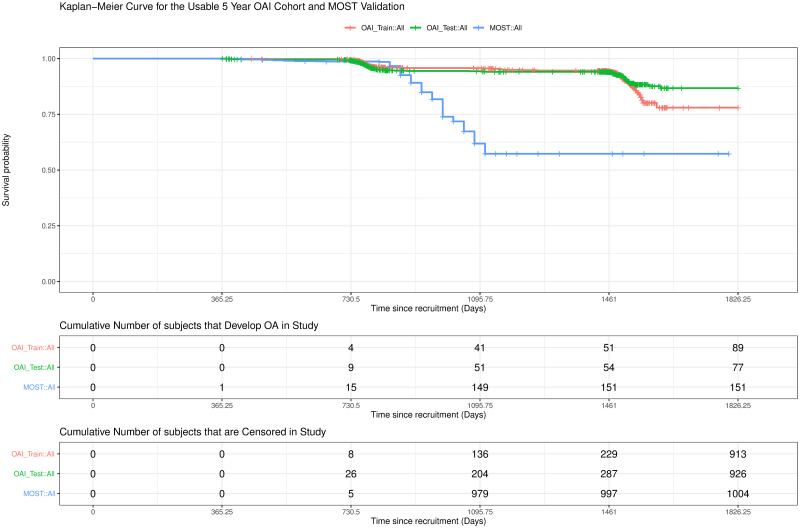
Observational KM curve stratified by sample. The red depicts the OAI training sample and the green shows the OAI test sample. The blue curve is the MOST validation data. The tables below illustrate the way in which the data is split between the samples.

As the data for the prognostic model differs from that in the diagnostic model, the training and test sets are also different. The training set and test set comprise of 1002 and 1003 subjects respectively. The external validation set contains n = 1155 subjects. The Kaplan-Meier curve in Fig 3 shows lines that represent the full cohort, training, test and MOST validation data. This shows that the training and test samples are a reflection of the whole cohort.

To ensure the modelling of the variables is appropriate for the assumptions made about proportional hazards, testing is carried out. The results from these investigations show that all of the covariates, along with the model as a whole, follow the proportional hazards assumption.

The next step in the analysis is to see if there are groups within the cohort, displaying different risk profiles. For example, to determine if there is a high and low risk group, and to establish what the criteria is for inclusion in each group. To stratify the group into cohorts the first step is to establish a cut point that gives the biggest separation in the subjects.

**Fig 4:**
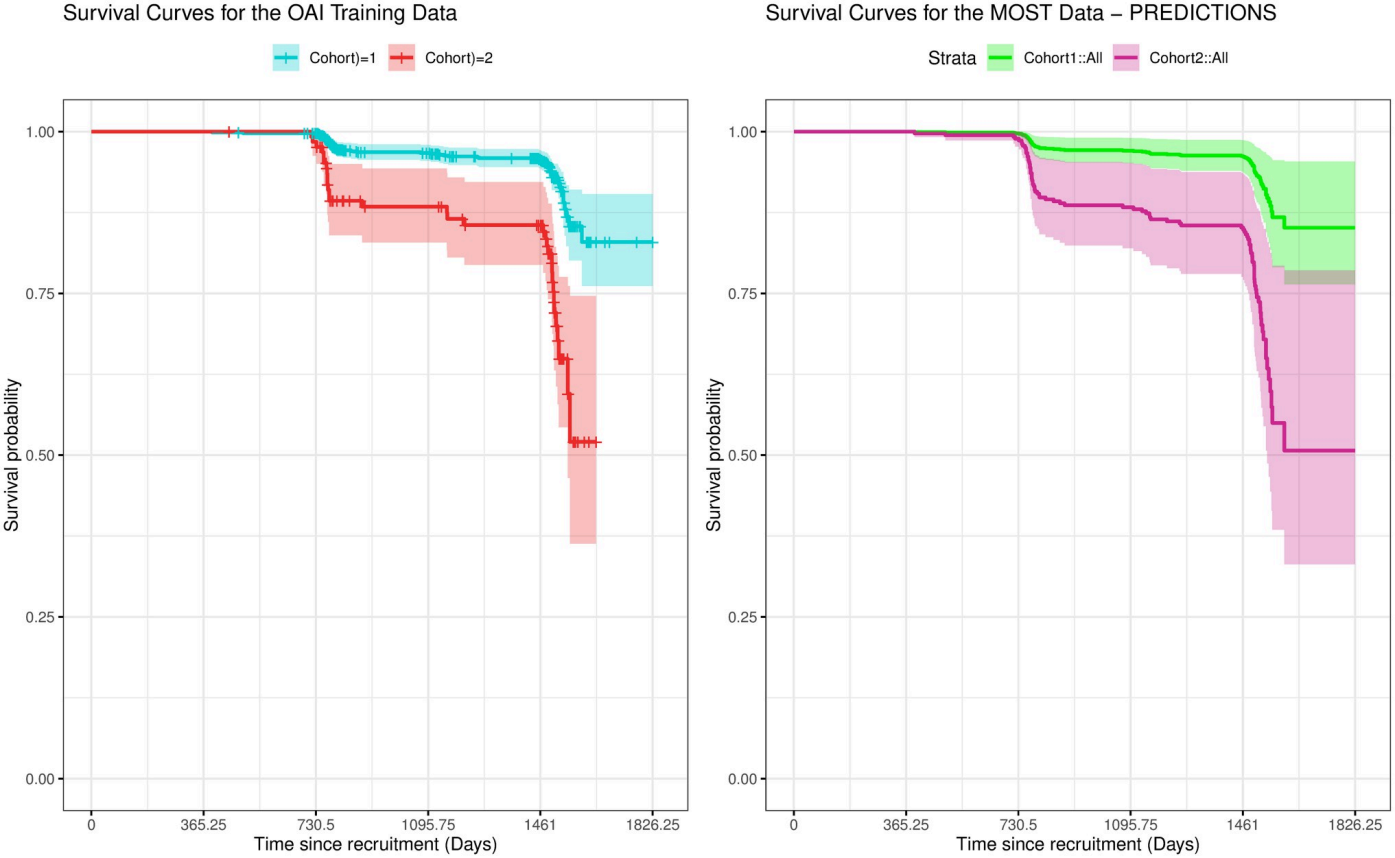
Risk stratification curves. Stratification curves on the left showing OAI training data showing the high and low risk cohorts. Note the last event recorded in cohort 2 within the 5-year span is at day 1642. The stratification curves on the right are the validation data showing the high and low risk cohorts fitted to the models developed using the OAI data.

Fig 4 shows the stratification curves on the OAI training data for the raw data and the predictions produced on the MOST validation. The last event recorded in cohort 2 on the training data within the 5-year span is at day 1642. The stratification curves produced are well separated with no crossover on the confidence intervals, which indicates that on unseen data the well-separated groups hold true.

**Fig 5:**
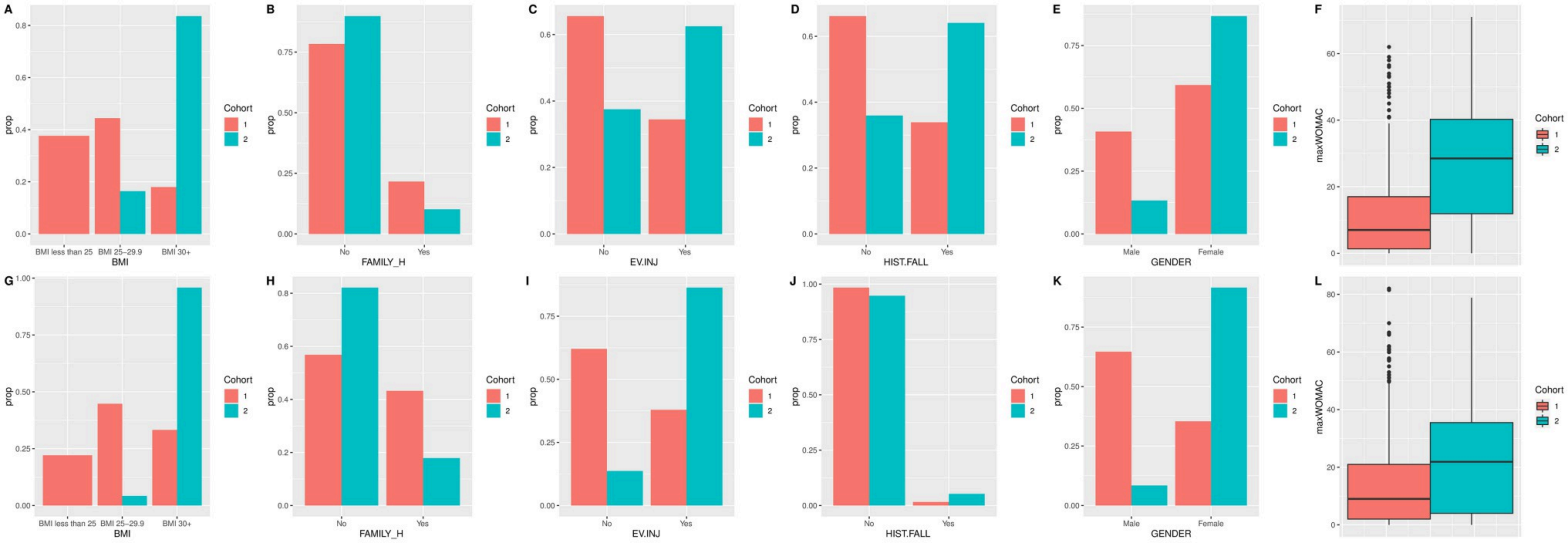
The cohort profiles per variable for the different strata. The red bars show cohort 1 and green show cohort 2. This representation of the profiles is the proportion of the group in each data category per cohort, graphs A-F are for the training set, and G-L are for the validation data.

For the model to have clinical value, the findings of the two risk cohorts need to be translated into human terms. For example, how the features influence that individual in relation to which risk group they will belong. The proportions are shown in Fig 5. The proportion plots are useful as they can be used easier to profile the groups in each cohort. For example, in Cohort 2 the majority of the subjects are female, all with a BMI over 25, and the majority have had previous knee injuries and a history of falling. The majority of the people in Cohort 2 have no family history of knee problems, which could mean that those who were aware of the issues with their family history of OA had already made changes to their behaviours and this helped with prevention or delay in developing KOA. When calculated, the AUC for the survival analysis for the OAI test set is 0.74 (0.7325–0.7439) and that of the MOST data is 0.72 (0.7190–0.7373).

**Fig 6:**
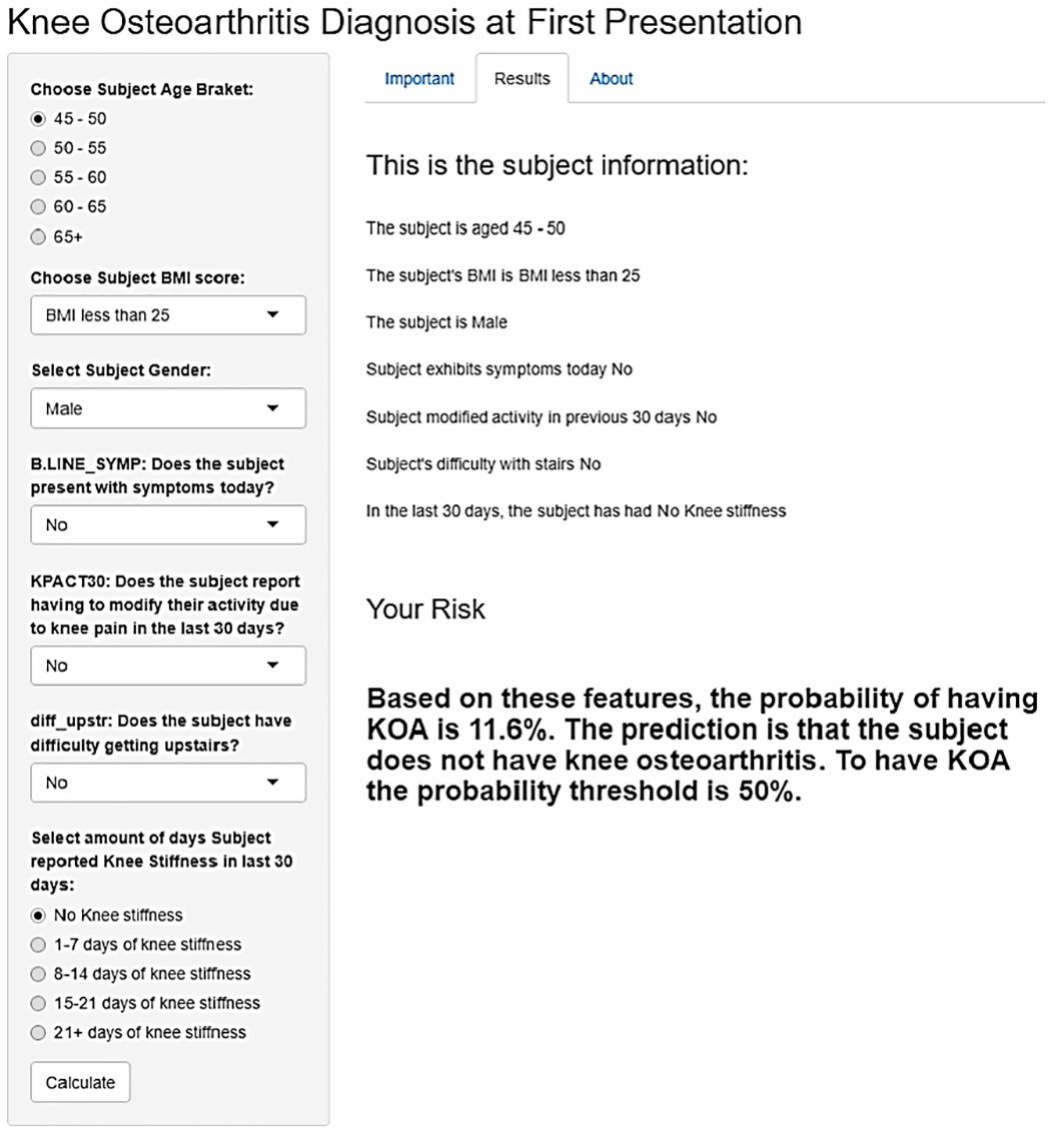
Diagnostic web app interface built in R Shiny. The app has multiple-choice options to allow the user to input variables that provide a probability of the participant having KOA based on the provided symptoms. This app was built using R Shiny [33].

**Fig 7:**
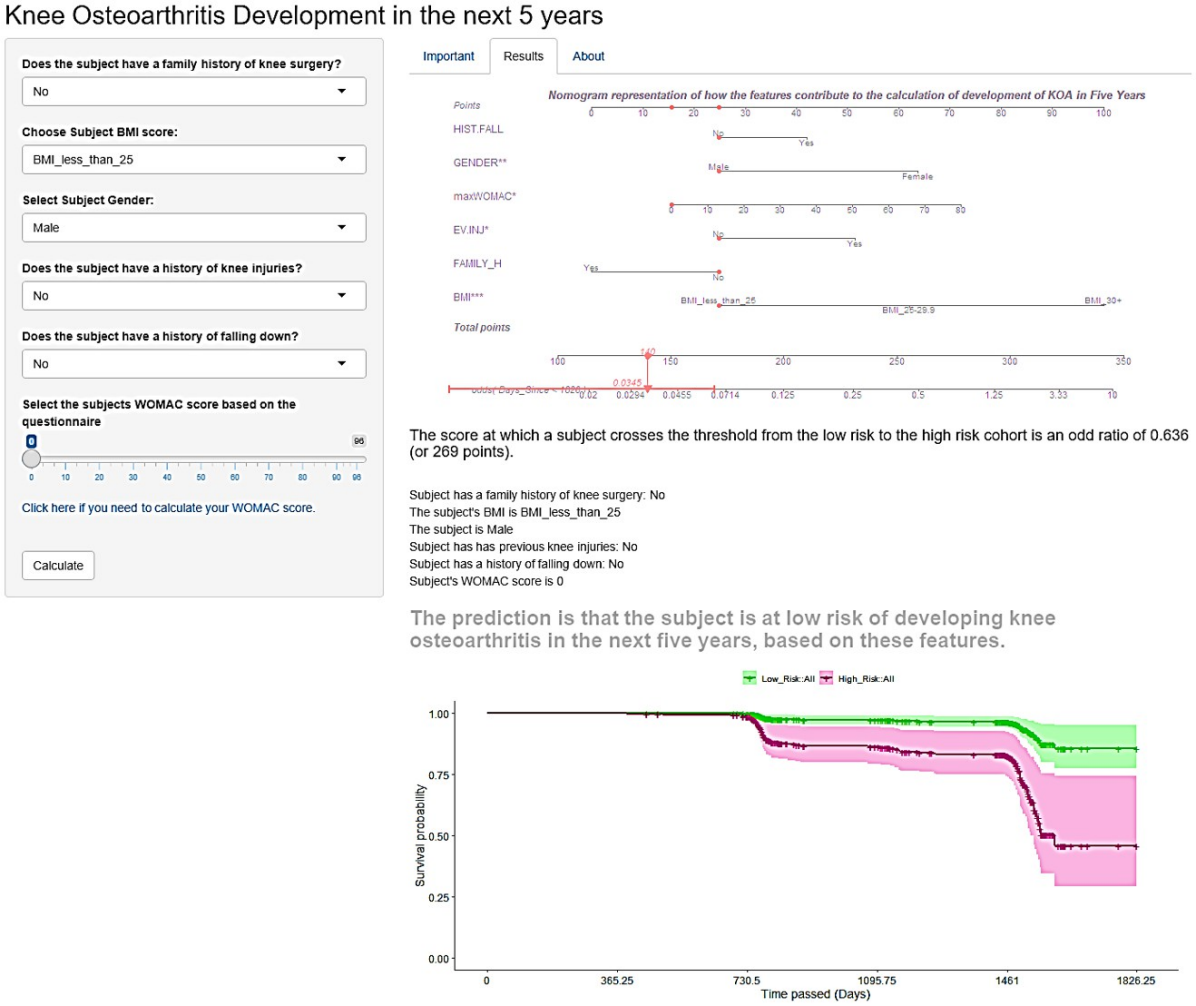
Prognostic app interface built in R Shiny. The app has multiple-choice options that relate to symptoms linked to the progression from a disease free state to KOA onset. The app also includes the option to link to the WOMAC questionnaire, should the participant not know their score. This app was built using R Shiny [33].

For the prognostic and diagnostic OA prediction models to be useful in a clinical setting they need to be user friendly, and implemented in a digital format such as a web based app. Both the diagnostic and prognostic apps are shown in Figs 6 and 7 respectively. At present, the apps are not yet publically available as they were originally produced as part of the OActive project as a prototype of an application that could be used within a clinical setting and as such are working towards making the app publically available through negotiation with OActive project leaders.

## Discussion

Both the prognostic and diagnostic models stood up to external validation with the MOST data. This helps to ensure that the model has not overfit to the training data. By having data in the OAI study come from different centres and using validation data that was collected from different sources, we have greatly reduced any chance of the model overfitting to the noise in the data. The approach of using observational features to determine the probability of presence and likelihood of onset of KOA has not been considered before in this way, as this approach uses a selection of variables from different domains about an individual.

Data from the real world often has missing values. There are many approaches to dealing with missing values, each having its own merits and disadvantages. There are two primary ways of dealing with missing values—deletion or imputation [40]. Commonly used approaches include imputation with forward and backward filling, multiple imputation and complete case analysis. Another approach is to use analytical methods that can deal with missing values [41].

Missing data causes issues when modelling, such as making handling the data and analysis difficult, reducing efficiency in models and introducing bias [42]. In any method of dealing with missing values there is bias introduced into the data, so the type of imputation used in any given analysis may be chosen for what works best for the given dataset [43].

Imputation uses the available data to fill in missing values. Although this is a commonly used approach, forward and backward filling are known to increase bias and potentially lead to false conclusions as data will artificially have repeated measures, and are not often recommended. Mean substitution replaces missing values with the mean of that variable, without altering the sample mean for the variable. However, mean imputation reduces the correlations involving the variables that are imputed. This approach has some good points for univariate analysis but poses problems if considering this approach within multivariate analysis [44].

Multiple imputation, most commonly multiple imputation by chained equations (MICE), is designed for missing at random data but can be extended to cases where data are missing not at random [45]. However, MICE can encounter problems in data with a large amount of observations and complex features like nonlinearities and high dimensionality. It also poses the additional problem of being difficult to implement, where single imputation and complete case analysis are easier to implement [43]. In any imputation method there is the potential for data leakage, which can impact the way the data performs in models and can impact the accuracy of predictions.

The final approach for dealing with missing values is complete case analysis [46]. This is the most common way of dealing with missing values. Complete case analysis works by removing cases where there are missing values present; as a result, this approach reduces the sample size. One disadvantage of this approach is that if the data are not missing completely at random then removing instances with missing data will introduce bias [47].

The data are used in a complete case format for modelling, with some imputation by marginalisation used for the validation dataset. Each imputation method adds bias, but the complete case analysis is easy to implement and straightforward, giving reason why it is the most popular method when dealing with missing values, despite its disadvantages.

The use of decision support tools in clinical situations has filtered into many different areas. A 2012 review showed that the implementation of decision support systems were greatly effective at improving the processing for which they were created [48]. Decision support tools are used frequently when related to cancer. For example, Adjuvant! is a computer program developed in 2001 to allow health professionals and patients to make informed decisions about treatment [49]. This application was for use once a patient had a cancer diagnosis but helped to provide useful insight for the patient into the steps involved in decision making related to their care. A similar application was produced by the International Ovarian Tumour Analysis. This app is for clinician use, helping to determine if a tumour is benign or malignant. There have been two versions of this app with different rules created. One version was created in 2008 and uses six predictors in the model [50]. The later version of the app, from 2016, calculates a risk of malignancy in tumours, using the original model as a base that was modified [51]. Apps such as those, including the one developed for KOA in this work, (see Figs 6 and 7) can be used in GP practices when a subject has symptoms or as part of screening to help educate the subject about their risk.

One of the aims throughout this work was to make a model that was interpretable and easy to use in a clinical environment. In order to do this, interpretable approaches were used in the modelling of the data and web based applications were developed for accessible use. These user interfaces offer the potential for the tool to double as a clinical aid and a resource for patient education. Having the risk model displayed in this way allows the patient to easily see and understand the way in which the factors relating to their life impacts on their individual risk of having or developing KOA. If the tools were to be used as a clinical aid they may help to improve patient flow from query to diagnosis and better allow for more in depth investigations, such as targeted x-rays for those who are on the borderline of having KOA or those at high risk. Another way that the interfaces could serve with clinical use is for patient signposting. Currently, the NHS offer lung screening visits to people aged between 55 and 74 who smoke or have previously smoked [52]. This initiative helps to detect lung conditions, such as cancer, earlier than they would have been picked up, allowing for better disease outcomes and more targeted treatments. Having a similar tool in place for a GP to use for KOA may help identify those at high risk of developing the disease in the next five years and allow for more targeted advice to those individuals, potentially having a positive outcome in relation to delaying the onset of KOA.

When looking at the area of knee osteoarthritis, survival modelling has predominantly focused on progression from an arthritic state to joint replacement. One example of this examined the importance of cartilage defects in older adults in relation to progression to knee replacement [53]. A similar study investigated the incorporation of radiographs when predicting the likelihood of total knee replacement within 9 years and the final Kellgren-Lawrence grade [54]. Some studies focus on the likelihood of developing KOA following certain treatment courses. For example, one such study examined the risk of requiring knee replacement surgery following treatment with intra-articular corticosteroid injections [55]. A similar study found that the use of intra-articular corticosteroid injection increases the risk of KOA progression [56]. Joint space narrowing was also studied as an outcome in survival modelling in patients with known symptomatic OA [57] showing that once radiographic changes were visible then the risk of progression in OA was significant. This is where our prognostic modelling approach differs, as it takes the subjects with no initial KOA and identifies those at a higher risk of developing the disease in a five-year follow-up window. This offers the chance to target healthy, at-risk, individuals before the onset of KOA, and delay the onset of disease, thus having the potential to reduce costs to the healthcare providers as treatment interventions may not be required as frequently as a result of educating the individual about their risks.

We have developed two models: one for diagnosis of radiographic KOA and the other for progression from a disease free state to radiographic KOA in a five-year time span. The models were developed specifically for radiological KOA, assigned by a clinician with a KL grade. When modelling we have removed the potential for repeated measures as our model considers only the worst case for symptoms and outcomes. We also removed the risk of bias by imputation through having a complete cases analysis. The progression model defines a high and low risk cohort for developing KOA over a five-year window. While we considered KOA as a binary variable, future modelling could consider more granular changes in KL grade. Finally, when looking at the model performance, the 2016 model from [12] uses ANN and LogR, and on the externally validated data their ANN AUC is the same as our LogR AUC, which outperforms the AUC of their externally validated LogR model [12]. Also worth noting is that both their model and our models’ internal AUC scores were roughly equivalent for our model and the PLOS One model [12].

LogR calculates the probability of an event happening based on factors in the model. A Cox model uses the factors as explanatory variables and uses those to explore the disease-free survival of a subject over a given time. Unlike LogR, a Cox model is dependent on time, meaning the hazard of an event happening changes with time. In this paper both LogR and Cox regression models are used to answer different questions- LogR the diagnostic problem and Cox the survival problem.

This study into KOA prediction in patients without disease poses the benefit of being the first of its kind. The diagnostic work builds on models by Losina et al [15], Joseph at al [21], Yoo et al [12] and Zhang et al [58], to produce an app that can be used to determine the chance of having KOA at the time of screening with only the use of clinical features. The prognostic model stratifies the population into high and low risk cohorts for developing KOA in a five year window. The models however are limited due to availability of data and the elements of missingness present.

The diagnostic model described in this paper could be seen as a primary care aid. To further expand this to be able to differentiate between unilateral and bilateral KOA the model would need to leverage information available in x-ray images. This expansion on the model would then rely on information that would typically be provided at a secondary care level, such as a hospital. The expanded model would be best suited to aid the clinician in assessing, from the x-ray, whether a person had KOA, and if so whether it was unilateral or bilateral.

Although there is a clear clinical need for tools to help diagnose and predict the risk of KOA, the models developed and described in this paper are a preliminary step toward a version that could be used within NHS clinical practice. The models developed use data from the US where the population demographic is different from the UK, which would be the target audience from these models, posing one limitation of the work. In order for these models to be used in the UK they would require validation on UK based data to ensure the features are still relevant given the different population demographic. From this point, the options relating to modelling for the UK would be to remodel entirely on the UK data or to use multitask learning to enrich the data sources with the aim of producing a more generalised model, suitable for both demographic cohorts. This would be a useful way to check that models built with different demographics do translate to different populations. This would also externally validate the model for the UK population.

## Conclusion

We have successfully created interpretable models, with app interfaces, for both diagnostic and prognostic purposes relating to KOA from features collected at first presentation. The use of interpretable methods to develop the models, combined with the added insight from the apps mean that it is clear how the message from the models can be translated from a high level to a patient level of understanding for maximum benefit.

The models built using the OAI data perform well when tested on the unseen MOST data and have comparable results with several other models already in existence that use more complex methods. The work using a Bayesian network approach had a model AUC of 0.78, the LogR model used in the paper that also used ANNs had a performance of 0.76 and 0.63 for the testing and validation data respectively [13, 59]. The same paper reported the performance of the ANN as slightly higher, 0.81 and 0.67 for the testing and validation data respectively. In comparison, the diagnostic model in this paper had an AUC of 0.75 for the test data and 0.67 for the validation data. The performance from this model is comparable to those from more complex approaches using more variables that that in the model reported here. The features that are used in the diagnostic model are related to the holistic view of the disease, such as knee swelling, knee pain limiting activity in the month prior to the visit, difficult navigating stairs and knee stiffness in the past 30 days along with BMI, gender and age.

Both the diagnostic and prognostic models have been converted into web apps so that they are more user-friendly for clinical settings. Apps such as these have been beneficial in other areas, such as ovarian cancer and the IOTA app [51]. With the clinical implementation of decision support tools, such as those we have created, there is potential to improve clinical management of patients from first presenting with KOA symptoms or disease management with the outlook to patient education.
